# miR-203 Regulates Cell Proliferation through Its Influence on Hakai Expression

**DOI:** 10.1371/journal.pone.0052568

**Published:** 2012-12-20

**Authors:** Vanessa Abella, Manuel Valladares, Teresa Rodriguez, Mar Haz, Moisés Blanco, Nuria Tarrío, Pilar Iglesias, Luís A. Aparicio, Angélica Figueroa

**Affiliations:** 1 Translational Cancer Research Group, Instituto de Investigación Biomédica A Coruña (INIBIC), Complexo Hospitalario Universitario A Coruña (CHUAC)-SERGAS, A Coruña, Spain; 2 Medical Oncology Unit, CHUAC-SERGAS, A Coruña, Spain; 3 Clinical Trials Service, Hospital Universitario Marqués de Valdecilla, Santander, Cantabria, Spain; 4 Pathology Department, CHUAC-SERGAS, A Coruña, Spain; Emory University School of Medicine, United States of America

## Abstract

Gene expression is potently regulated through the action of microRNAs (miRNAs). Here, we present evidence of a miRNA regulating Hakai protein. Hakai was discovered as an E3 ubiquitin-ligase that mediates the posttranslational downregulation of E-cadherin, a major component of adherens junctions in epithelial cells and a potent tumour suppressor. Recent data have provided evidence that Hakai affects cell proliferation in an E-cadherin-independent manner, thus revealing a role for Hakai in the early stages of tumour progression. Furthermore, Hakai is highly up-regulated in human colon adenocarcinomas compared to normal tissues. However, the molecular mechanisms that regulate Hakai abundance are unknown. We identified two putative sites of miR-203 interaction on the Hakai mRNA, in its 3′-untranslated region (UTR). In several human carcinoma cell lines tested, overexpression of a miR-203 precursor (Pre-miR-203) reduced Hakai abundance, while inhibiting miR-203 by using an antisense RNA (Anti-miR-203) elevated Hakai levels. The repressive influence of miR-203 on the Hakai 3′-UTR was confirmed using heterologous reporter constructs. In keeping with Hakai's proliferative influence, Anti-miR-203 significantly increased cell number and BrdU incorporation, while Pre-miR-203 reduced these parameters. Importantly, the growth-promoting effects of anti-miR-203 required the presence of Hakai, because downregulation of Hakai by siRNA suppressed its proliferative action. Finally, *in situ* hybridization showed that miR-203 expression is attenuated in colon tumour tissues compared to normal colon tissues, suggesting that miR-203 could be a potential new prognostic marker and therapeutic target to explore in colon cancer. In conclusion, our findings reveal, for the first time, a post-transcriptional regulator of Hakai expression. Furthermore, by lowering Hakai abundance, miR-203 also reduces Hakai-regulated-cell division.

## Introduction

Carcinoma arises from epithelial cells on which cancer cells start an uncontrolled proliferation and, in order to metastasize, some cells detach from the primary tumour, migrate and invade through tissues. One hallmark of metastasis is the disruption of epithelial integrity and loss of intercellular adhesion. Downregulation of cell–cell adhesion is characterized by the loss of E-cadherin, the best protein characterized and prototype member of the classical cadherins in epithelial cells, which are potent tumour suppressors in epithelial cells [1]. Epithelial tumours often lose E-cadherin partially or completely as they progress toward malignancy [2], [3]. Given the great impact of E-cadherin in cancer, the mechanisms that control E-cadherin inactivation in human cancers have been extensively studied [4], [5]. In 2002, the protein Hakai was identified as the first post-translational regulator of E-cadherin stability. Since then, many studies on the emerging biological functions of Hakai have underscored its influence on tumour progression and disease [6]. Hakai is an E3 ubiquitin-ligase that mediates the ubiquitination of E-cadherin protein upon Src activation, in turn mediating its lysosomal degradation [6]–[9]. Since then, novel proteins substrates for Hakai have been identified, such as Cortactin, a protein critically involved in the reorganization of actin cytoskeleton in cell protrusions, and DOK1, which binds to p120-rasGAP, a potent inhibitor of Ras oncogene [10]. Besides influencing cell adhesion, Hakai has also been implicated in controlling cell migration and embryogenesis [11]–[13], and it can control cell proliferation in an E-cadherin-independent manner, further supporting a role for Hakai in early stages of tumorigenesis [14], [15]. Accordingly, Hakai is highly up-regulated in human colon adenocarcinomas compared to normal tissues. Underscoring the interest exploiting Hakai as a therapeutic target, its molecular structure was recently solved [10]. However, to-date, no regulators of Hakai expression have been described.

Over the last decade, microRNAs (miRNAs) have emerged as key players in carcinogenesis. Aberrant expression of miRNAs has been demonstrated to play a critical role in the initiation and progression of several cancers [16]. miRNAs are small (∼22-nt), single-stranded, non-coding RNAs that play a key role in development and diseases through post-transcriptional regulation of gene expression [17]–[19]. Synthesized as longer primary transcripts by RNA polymerase II, pri-miRNAs are processed by the nuclear RNase Drosha into 70-nt hairpin precursor miRNAs (pre-miRNAs). Following Exportin 5-mediated transport to the cytoplasm, pre-miRNAs are further processed by the RNase Dicer, giving rise to mature miRNAs that assemble with members of the argonaute (Ago) protein family into the RNA-induced silencing complex (RISC). The miRNA then directs the complex to target mRNAs typically reducing their translation and/or stability [20]–[24]. miRNAs generally bind with partial complementary one or more sites in the target 3′-untranslated region (3′UTR) of the target mRNA [25].

Here, we describe the identification of miRNA-203 as a negative regulator of Hakai expression. Our results demonstrate that miR-203 targets Hakai mRNA by binding to the 3′UTR of the Hakai mRNA, lowering Hakai expression and decreasing cell proliferation. Furthermore, immunohistochemcal analysis revealed that Hakai protein levels were higher in paired colon cancer tissues compared to adjacent healthy colon tissues and an inverse correlation was found for miR-203 levels by *in situ* hybridization, further suggesting a tumour suppressor role for miR-203 in colon cancer.

## Materials and Methods

### Antibodies and Materials

The rabbit polyclonal anti-Hakai antibody (Hakai-2498) and the pEGFP-Hakai construct were kindly provided by Dr. Yasuyuki Fujita [14]. Antibody anti-AKT2 was from Santa Cruz Biotechnology (Santa Cruz, CA). Anti-GFP was from Abcam (Cambridge, UK), anti-α-tubulin antibody was from Sigma-Aldrich (St Louis, MO), anti-BrdU antibody was from Calbiochem (Darmstadt, Germany), HRP-rabbit and mouse polyclonal antibodies were from GE Healthcare (UK) and E-cadherin antibody was from Invitrogen. All antibodies were used at a dilution of 1∶1000 for Western blot analysis, except the anti-GFP antibody, which was used at a dilution of 1∶5000. Cells were transiently transfected with small RNAs and/or plasmids using Lipofectamine 2000 (Invitrogen, UK), following the manufacturer’s instructions. At 48 h after transfection, cells were processed for RNA and protein analysis. The pre-miRNA and anti-miRNA for human miR-203 and miR-21, and scrambled negative controls (Ctrl) miRNA were obtained from Life Technologies (Applied Biosystems, UK) and used at final concentration of 3 µM. Oligos used for Hakai siRNA were Hakai-1 (CTCGATCGGTCAGTCAGGAAA) and Hakai-2 (CACCGCGAACTCAAAGAACTA) as previously described [14] and as a negative control (Ctrl), we used scrambled siRNA from Sigma-Aldrich (St Louis, MO).

### Cell Lines and Human Tissue Samples

Human cell lines HeLa (cervical carcinoma), HEK-293 (embryonic kidney cells), HT29 (colorectal adenocarcinoma), SW480 (colon adenocarcinoma), SW620 (colon adenocarcinoma) and A549 (lung adenocarcinoma) were obtained from American Type Culture Collections (Manassas, VA). Cells were cultured in DMEM or RPMI containing penicillin/streptomycin and 10% FCS at 37°C and ambient air supplemented with 5% CO_2_. Formalin-fixed, paraffin-embedded (FFPE) colon cancer tissues were obtained from the pathological anatomy department from the Complejo Hospitalario Universitario A Coruña (CHUAC, A Coruña), under informed consent from all the patients and research investigation was approved by the institutional research ethics committee. The data were analyzed anonymously. All slides were reviewed by a pathologist expert for the identification of the tumour region as well as adjacent normal colon epithelium.

### RNA Analysis and Protein Analysis

Total RNA was isolated using TriPure Reagent (Roche, Germany) according to the manufactureŕs instruction. After isolation, the obtained pellet was washed by following an alternative protocol described for small RNAs in RiboPure (Life Technologies, UK). The quality and quantity of the obtained RNA was determined by using Nanodrop ND-spectrophotometer (Thermo Fisher Scientific, MA, USA). After reverse transcription (RT) using random hexamers and SuperScript first-strand Synthesis System for RT-PCR (Invitrogen, UK), real-time quantitative PCR analysis was performed using gene-specific primers 5′-CGCAGACGAATTCCTATAAAGC -3′ and 5′-CCTTCTTCATCACCAGGTGG -3′ for human Hakai; and 5′-TGACCTTGATTTATTTTGCATACC-3′ and 5′-CGAGCAAGACGTTCAGTCCT-3′ for HPRT. PCR was performed by using Light Cycler 480 SYBR Green I Master (Roche, Germany).

For miRNA analysis, 25 ng of the extracted RNA was amplified and detected by using the Taqman microRNA detection assay following the manufacturer’s procedure (Applied Biosystems, UK). Probes for miR-203 and miR-21 (Applied Biosystems, UK) were used and U6 snRNA probe was employed for normalization. The amplification and quantification of cDNA was carried out by using a LightCycler 480 real-time lightcycler (Roche, Germany).

For protein extraction, cell lysates (30 µg of proteins) were obtained by using 1% Triton X-100 lysis buffer (20 mM Tris/HCl pH 7.5, 150 mM NaCl and 1% Triton X-100) containing 5 µg/ml leupeptin, 50 mM PMSF and 7.2 units of trypsin inhibitor aprotinin and western blotting was performed as we described previously [26].

### EGFP Reporters Containing 3′-UTR Hakai mRNA Sequence

To construct the EGFP reporter plasmid containing 3′UTR or Hakai mRNA we used the following PCR primers: 5′- GGTTCCCTCGAGCTAAGGAAGAGTACCTCTTATCGAGG-3′ and 5′-GGTTCCGAATTCCCTCAACATTTCAGTGCC-3′ and the resulting PCR products were ligated into pEGFP (Clontech, California, USA). The reporter vector and the pEGFP alone were cotransfected with the indicated miRNAs as described above. Cells were fixed with 3.7% paraformaldehyde/PBS for 15 min. Immunofluorescence images were analyzed by epifluorescence microscopy. Phase contrast images were acquired using a Nikon Eclipse-Ti microscope.

### Proliferation Assay and Flow Cytometry Analysis

For quantification of cell numbers, 2.5×10^4^ cells were plated per well into a 6-well plate; 48 h after transfection, cells were counted using hemocytometer. For colorimetric analysis, 1×10^4^ cells were plated per well into a 96-well plate and after 48 h of transfection they were treated with 10 µM BrdU for 2 h. BrdU incorporation into newly synthesized DNA was measured using a cell proliferation colorimetric immunoassay kit (Calbiochem, Germany) according to the manufacturer’s instructions. For the flow cytometry analysis, cells were trypsinized, washed with PBS containing 1 mM EDTA, and fixed following incubation in PBS containing 100 µg/ml of RNase and 50 µg/ml propidium iodide (Sigma), DNA was analyzed by FACSscan flow cytometer (Becton Dickinson, USA). Data were acquired and analyzed by Cell Quest Program (Becton Dickinson, USA).

### Immunohistochemistry and *in situ* Hybridization

For immunohistochemistry, tissue sections were deparaffinized and antigen retrieval was performed in citrate buffer (Dako REAL, Denmark) by heating the samples (2100 Retriever; PickCell Laboratories). After 20 minutes at room temperature, the endogenous peroxidase activity was inhibited with peroxidase-blocking solution (Dako REAL, Denmark). Primary antibodies were incubated overnight at 4°C. Finally, detection was carried out by using Dako REAL EnVision Detection system according to the manufacturer's instructions. Nuclei were slightly counterstained with Gill’s hematoxylin. Calibration and quantification of the images was performed with AnalySIS^D^ 5.0 software (Olympus Biosystems, Hamburg, Germany).

*In situ* hybridization was performed using the instruction of v2.0 miRCURY LNA microRNA ISH Optimization kit for FFPE (Exiqon, Denmark), using double DIG-labeled mercury LNAmicroRNA Detection probes. It was used miR-203 probe and LNA U6 snRNA for detection of control probe and scramble microRNA probe. All probes were used at final concentration of 0.25 nM except for U6, which was used at 0.005 nM.

### Target Prediction

miRNA target prediction was carried out by using TargetScan (release 5.1; http://www.targetscan.org/).

### Statistical Analysis

Unless indicated, all experiments were analyzed by using Students *t*-test to evaluate differences between treatments at the indicated significance levels. For quantitative image analysis of immunohistochemical staining, the Mann-Whitney U-test was employed. SPSS statistics 20 (IBM, New York, US) was used for data management in the statistical analyses.

## Results

### miR-203 Regulates Hakai Protein Levels

**Figure 1:**
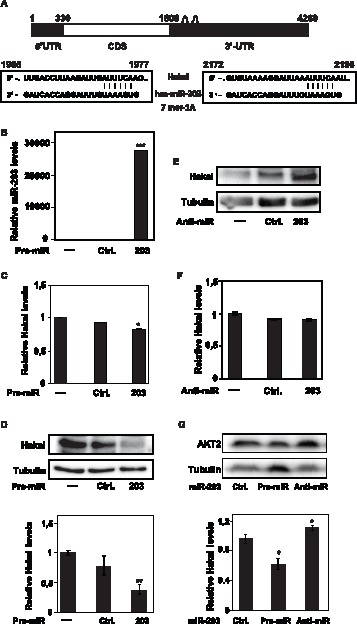
miR-203 modulates Hakai levels in HeLa cells. A, schematic representation of Hakai mRNA depicting two predicted miR-203 target sites within the Hakai 3′UTR. Alignment of consensus sequences of Hakai mRNA with miR-203; top strand, Hakai sequence; bottom strand, miR-203. B, miR-203 levels measured by RT-qPCR after 48 h of untransfected HeLa cells, transfection with scrambled control miRNA (Ctrl) or the precursor miR-203 (Pre-miR-203); U6 snRNA levels was used as control to monitor loading differences. C, levels of Hakai mRNA normalized to control HPRT mRNA were measured 48 h after transfecting HeLa cells with the indicated miRNAs. D, the effect of the indicated transfected miR-203 on Hakai levels in HeLa cells were tested in HeLa whole-cell lysates by Western blotting (*Top*) and quantified by densitometry (*Bottom*), using Hakai antibody and α-tubulin as loading control for normalization. E, the levels of Hakai and loading control α-tubulin were tested in whole-cell lysates by Western blotting 48 h after transfecting HeLa cells with the anti-miR-203 or scrambled control miRNA. F, the levels of Hakai mRNA 48 h after transfection of HeLa cells with the anti-miR-203, or scrambled control miRNAs (each normalized to HPRT levels), were analyzed by RT-qPCR. G, western blotting analysis (*Top*) and quantification by densitometry (*Bottom*) of AKT2 levels in HeLa cells expressing either pre-miR-203 or anti-miR-203, processed as described in D. Western blotting signals were quantified by densitometry and shown in 1D. Values in B–D and F–G are the means ± SEM from three independent experiments. Statistical analyses indicate the significant difference in the indicated transfected miRNAs with respect to transfected scrambled control (Ctrl) miRNA (*p<0.05, **p<0.01, ***p<0.001). The western blotting data are representative of three experiments.

In recent years, the refinement of miRNA-mRNA prediction algorithms, along with improved knowledge of miRNA target recognition and increasing availability of relevant data sets have greatly improved our ability to anticipate regulatory miRNA:mRNA interactions. Data driven algorithms rely on important discriminative features learned from data using sophisticated models [27]–[29]. miR-203 was predicted to bind to Hakai mRNA at two sites within 3′-UTR, positions 1965 to 1977 and from 2172 to 2198 (Targetscan) (Figure 1A). The effect of miR-203 on Hakai expression was tested in HeLa cells, which did not express E-cadherin, as shown in Figure S1 and previously published [30]. To elevate miR-203 levels, the precursor miR-203 transcript (pre-miR-203) was transfected and after 48 h after transfection, Hakai expression was analyzed by reverse transcription followed by quantitative real-time PCR (RT-qPCR) and Western blotting. The increased level of miR-203 was confirmed in cells transfected with pre-miR-203, compared to cell transfected with scrambled control miRNA (Ctrl) and to un-transfected cells (Figure 1B). Under these conditions, Hakai mRNA levels were only modestly lower (Figure 1C), while Hakai protein levels were strongly reduced in the pre-miR-203 group compared to the scrambled Ctrl miRNA and un-transfected groups (Figure 1D). In addition, 48 h after transfection of antisense transcripts to reduce the levels of miR-203 (anti-miR-203), Hakai protein levels were higher in the scrambled Ctrl miRNA and un-transfected cells (Figure 1E), again in the absence of changes in Hakai mRNA levels (Figure 1F), suggesting that miR-203 influences Hakai mRNA translation rather than its degradation. The effect of miR-203 upon Hakai levels was specific, as reduction or overexpression of other miRNAs that were not predicted to target Hakai mRNA, such as miR-21, did not influence Hakai protein levels (Figure S2). Finally, the effect of the transfected pre-miR-203 and anti-miR-203 in HeLa cells was also slightly seen on Akt2 (Figure 1G), a previously described target for miR-203 in bladder cancer [31], [32].

**Figure 2:**
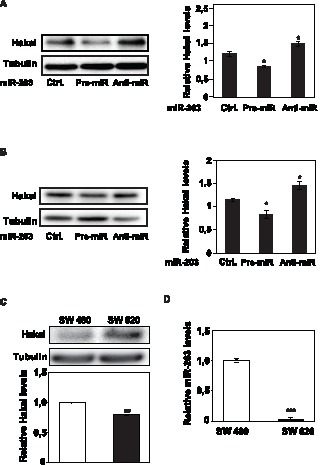
Influence of miR-203 on Hakai levels in several cell lines. A, the effect of modulating miR-203 levels in 293 cells was studied as described in 1D and 1E; Western blot analysis (*left panel*) and quantification by densitometry (*right panel)*. B, the effect of modulating miR-203 levels in A549 cells was studied as described 1D and 1E. Western blot analysis (*left panel*) and quantification by densitometry (*right panel).* C, endogenous Hakai expression levels in human colorectal cell lines (SW480 and SW620), as assessed by western blotting (upper panel) and by RT-qPCR (lower panel) analysis. D, endogenous miR-203 levels in human colorectal cell lines (SW480 and SW620) by RT-qPCR (lower panel). Values (A–D) are the means ± SEM from three independent experiments. Significant differences in the indicated transfected miRNA with respect to transfected scrambled control (Ctrl) miRNA for A–B, and the between SW480 and SW620 cell lines for C–D are indicated (*p<0.05, **p<0.01, ***p<0.001, n = 3). The western blotting data are representative of three independent experiments.

The levels of Hakai and miR-203 were monitored in other cultured human epithelial cell lines (human lung adenocarcinoma A549, and human embryonic kidney HEK293 cells). Transfection of HEK293 and A549 cells with anti-miR-203 similarly elevated Hakai levels, while pre-miR-203 lowered Hakai levels (Figure 2A and 2B, respectively). Hakai and miR-203 expression levels were also analyzed in SW480 and in SW620 colon cell lines (Figure 2C and 2D), two cell lines established from a primary colon adenocarcinoma and its lymph node metastasis, respectively. In this limited analysis, higher protein Hakai levels (but not Hakai mRNA levels) were detected in SW620 than SW480 (Figure 2C), further supporting the role of Hakai in tumour progression and malignancy in colorectal cancers. Finally, the detected higher Hakai protein levels in SW620 correlated with reduced miR-203 levels, while low Hakai levels in SW480 correlated to an increased miR-203 levels (Figure 2C and 2D). These data support the notion that miR-203 could broadly contribute to reduce Hakai expression.

### Hakai is a Direct Target of miR-203

**Figure 3:**
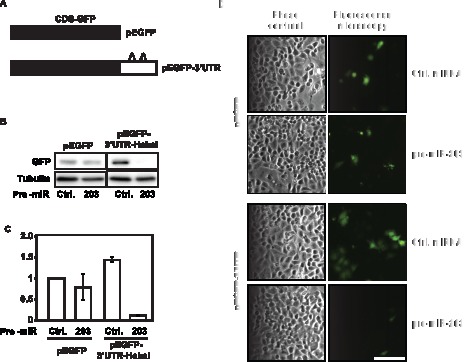
miR-203 influence on Hakai reporter construct. A, schematic of EGFP reporter constructs bearing either no Hakai mRNA sequences (pEGFP) or miR-203 target sites on the Hakai 3′-UTR (pEGFP-3′UTR). B, 48 h after cotransfection of the plasmids with scrambled control miRNA or Pre-miR-203, the level of EGFP expressed was analyzed by Western blotting. C, quantification of panel B showing the effect of scrambled Ctrl miRNA or pre-miR-203 on pEGFP and pEGFP-3′UTR. Significant differences in EGFP expression from pEGFP-3′UTR cotransfected with scrambled control miRNA (Ctrl) and pEGFP-3′UTR cotransfected with pre-miR-203 are indicated (***p<0.001). Western blotting data are the means ± SEM from three independent experiments.

To investigate whether miR-203 repressed Hakai expression through the predicted target sites on the Hakai 3′-UTR, we studied the influence of miR-203 on EGFP reporter constructs bearing the segments of the Hakai mRNA with the predicted mi-203 sites (Figure 3A). Expression of the parent control EGFP reporter (pEGFP) and the Hakai 3′-UTR miR-203 site (pEGFP-3′UTR) was tested in cells transfected with either scrambled Ctrl miRNA or pre-miR-203. By Western blot analysis (Figure 3B), miR-203 overexpression had no influence on EGFP expression from pEGFP, while it reduced EGFP levels expressed from pEGFP-3′UTR (down to ≈10% of the levels seen in the scrambled Ctrl miRNA group, quantification shown in Figure 3C). These findings were verified by fluorescence microscopy (Figure 3D), where EGFP fluorescence was markedly and selectively reduced after overexpression of pre-miR-203 in cells transfected with pEGFP-3′UTR. Taken together, these results indicate that the 3′UTR of Hakai mRNA contains sequences through which miR-203 directly represses Hakai expression levels.

### miR-203 Mediates Changes in Cell Proliferation through its Influence on Hakai Levels

**Figure 4:**
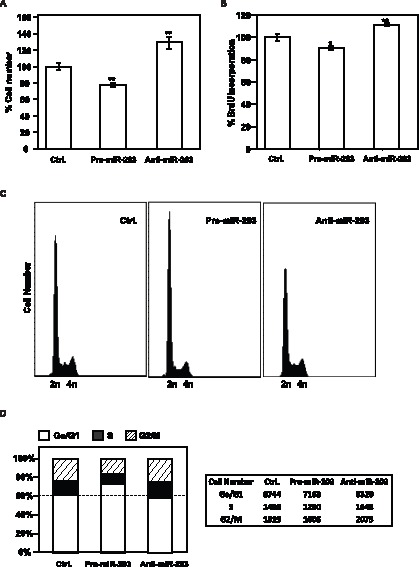
Influence of miR-203 on cell proliferation. A, 48 h after transfection of HeLa cells with the miRNAs shown, cell numbers were measured using a hemocytometer and represented as percentage of cells relative to the scrambled Ctrl miRNA group. B, measurement of BrdU incorporation by 48 h after transfection of HeLa cells with the indicated miRNAs. The data in panels A, and B are the means ± SEM from three experiments. Significant differences in cell number (A) and BrdU incorporation (B) in the indicated transfected miRNA compared to transfected scrambled (Ctrl) miRNA are indicated (*p<0.05, **p<0.01). C, forty-eight h after transfection with scrambled Ctrl miRNA, Anti-miR-203, or Pre-miR-203, HeLa cells were subjected to FACS analysis. D, the relative G1, S, and G2/M compartments shown in C were calculated and represented in left panel, and total cell numbers in every stage cell cycle compartment were included in the right panel. Data (C and D) are representative of three independent experiments.

Since it was previously described that Hakai could influence cell proliferation in an E-cadherin-independent manner, we used epithelial cells that did not express E-cadherin to further analyze cell division (Figure S1) [30]. In HeLa cells, we studied the effect of reducing and elevating miR-203 on cell proliferation, by measuring cell numbers 48 h after transfection. Pre-mir-203- transfected HeLa cells, which express low levels of Hakai protein (Figure 1 and 2), showed reduced cell numbers compared to scrambled Ctrl miRNA population, while transfection of anti-miR-203 increased Hakai protein levels (Figure 1 and 2), and cell numbers (Figure 4A). Measurement of BrdU incorporation further confirmed that cell proliferation was lower in anti-mir-203 transfected cells while it increases in pre-miR-203-transfected cells (Figure 4B). Other cell types were previously shown to be affected similarly following modulation of miR-203 levels [32]–[35]. In addition, flow cytometry analysis of cell cycle distribution further revealed that pre-miR-203-transfected population had the smallest S and G2/M phases and the largest G1 phase, while anti-miR-203-transfected cells had the largest proportion of S-phase and G2/M compartments (Figure 4C and 4D). Together with the changes in DNA replication and cell cycle distribution profiles, these results indicate that the miR-203 affected cell proliferation.

**Figure 5:**
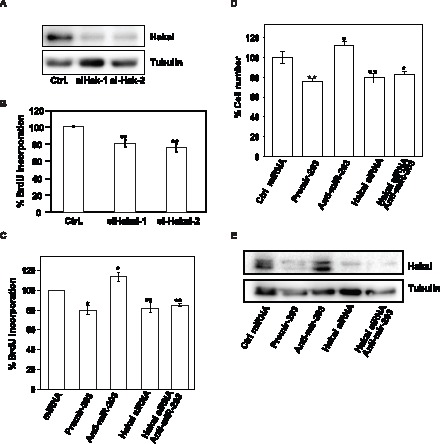
Influence of miR-203-regulated Hakai on cell proliferation. A, levels of Hakai and loading control α-tubulin were tested in whole-cell lysates by Western blotting 48 h after transfecting HeLa cells with two different siRNAs oligos for Hakai relative to the scrambled Ctrl small RNA group. B, 48 h after transfection of HeLa cells with the indicated siRNA oligos, cell numbers were measured by BrdU incorporation assay and represented as percentage of cells. C, measurement of BrdU incorporation 48 h after transfection of HeLa cells with the indicated small RNAs. D, measurement of cell number by hemocytometer after 48 h of transfection of HeLa cells with the indicated small RNAs. E, forty-eight h after transfecting HeLa cells with the indicated small RNAs, Hakai and α-tubulin levels were measured by Western blot analysis. Western blotting data are representative of three independent experiments. The data in panels B–D are the means ± SEM from three experiments. Significant differences in BrdU incorporation (B and C) and in cell number (D), compared to the transfected scrambled (Ctrl) small RNA are indicated (*p<0.05, **p<0.01).

To examine if the effects of miR-203 on cell proliferation were dependent on changes in Hakai abundance, we studied the effect of knocking down Hakai on the proliferation rate of cultured epithelial cells. Hakai protein were potently repressed following transfection of two different Hakai-specific small interference (si)RNA oligos (Figure 5A), an intervention that significantly decreased BrdU incorporation (Figure 5B). Importantly, the anti-miR-203-triggered increase in cell numbers was specifically dependent on the presence of Hakai, as concomitant Hakai silencing by siRNA in cells transfected with anti-miR-203 completely prevented the anti-miR-203-elicited proliferation, as shown by BrdU incorporation analyses and by counting cell numbers using hemocytometer (Figure 5C and 5D). As shown in Figure 5E, the increase in Hakai protein triggered by anti-miR-203 was abolished in cells co-transfected with siRNA-Hakai and anti-miR-203. Moreover, ectopically expressed pEGFP-Hakai, lacking its 3′UTR, was cotransfected together with the pre-miR-203, anti-miR-203 and scrambled control miRNA. Exogenous Hakai protein levels were not affected by the presence of either of two transfected miRNAs compared to scrambled control miRNA (Figure S3), in keeping with the notion that the effect was specifically dependent on the 3′UTR of Hakai. Taken together, our data support the view that miR-203 lowers cell proliferation at least in part by reducing Hakai protein levels.

### miR-203 and Hakai Expression in Tumour and the Adjacent Healthy Colon Tissues

**Figure 6:**
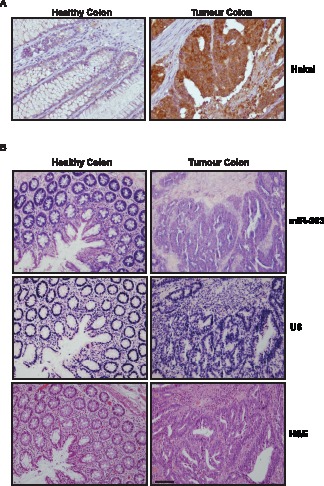
Hakai and miR-203 expression in colon tissues. A, immunohistochemical analysis of Hakai protein expression in normal colon versus colon cancer tissues. Differences between tumour samples compared to its paired healthy tissues are statistically significant (***p<0.001, n = 19). B, *in situ* hybridization (ISH) for miR-203 (upper panel) and U6 snRNA (as control probe, middle panel) expression in colon cancer tissues compared to normal colon tissue. Haematoxylin and eosin (H&E)-stained section (lower panel) was included to identify tumour area. Scale bar, 200 µm.

Given our earlier observations that Hakai levels were higher in human colon cancer tissues [14], we extended this analysis to 19 pairs of human tumour and non-tumour colon samples. Hakai abundance was markedly higher in tumour samples compared to adjacent healthy colon tissues, as shown in a representative image Figure 6A. The scoring result of the quantification of the intensity signal mean per area in healthy and its paired tumour samples is shown in Figure S4. Quantitative image analysis of immunohistochemical staining revealed that tumour samples were significantly more intensely stained for Hakai than its paired healthy samples, by Mann-Whitney U test (*** = p<0.001, n = 19).

To study if miR-203 levels correlated inversely with Hakai expression levels, we assessed miR-203 abundance by in *situ* hybridization in four paired samples (Figure 6B). miR-203 shows an attenuated expression in three of colon tumour tissues compared to normal colon tissues and no differences were detected in the other case analysed (data not shown) or when using control U6 probe (Figure 6B, middle panel). Taken together, these results suggest that reduced miR-203 levels in cancer tissues could contribute to the up-regulation of Hakai levels. Our findings support the notion that miR-203 function as a tumour suppressor [35], [36].

## Discussion

In this study, we describe the repression of Hakai expression by miR-203. Our findings indicate that miR-203 mainly reduces Hakai protein levels, with only a slight effect on Hakai mRNA levels, suggesting that miR-203 controls Hakai translation, in keeping with the mechanisms of miRNA-mediated repression of translation [24], [37]. Gene silencing by miRNA may occur by mRNA degradation and/or repression of translation. The fate of the target mRNA appears to be decided by the extent of base-pairing to the miRNA. If there is a complete or near-perfect complementation between the miRNA and target mRNA sequence, Ago2 can cleave the mRNA and lead to direct mRNA degradation. On the other hand, the presence of multiple, partially complementary sites in the target mRNA will preferentially inhibit protein translation without strongly affecting mRNA levels. However, this distinction is still controversial, as even in the latter situation, miRNAs can strongly decrease mRNA levels [20], [38]. Ectopic reporters containing the 3′UTR of Hakai protein showed that miR-203 elicited its repressive influence at least in part through the Hakai 3′UTR, which contains two miR-203 *in silico* predicted sites (Figure 3A). We have used TargetScan to predict miRNA targets based in seed sequence conservation [38], [39]. The degree of conservation of the binding site is determined by the number of species with the same sequence and/or by the phylogenetic distance between the species sharing the same sequence. miR-203 belongs to miRNA families poorly conserved among mammals or vertebrates. Two predicted targets sites are found into the first five-hundred pair bases of the 3′UTR of Hakai mRNA, the region most likely to bear miRNA regulatory sites. By Targetscan search (version release 5.1), the type of predicted sites for miR-203 was 7mer-1A (a match to positions 2–7 of the mature miRNA, the seed sequence, followed by an ‘A’). The negative score of the site-type contribution is associated with a more favourable site (for both sites the site type contribution is −0.074) [29], and the position contributions are −0.032 for the site closest to the coding region, and −0.008 for the second one, indicating that the first one is associated to a more favourable binding site for regulation. By searching for Hakai in other data base such as Tarbase 6.0 [40], miR-26b-5p miRNA was found to associate to Hakai, although this association was only described as a result of a high-throughput microarray screening, and the functional role of this interaction is still unknown [41]. In conclusion, up to date, miR-203 is the first validated miRNA to influence Hakai protein levels.

Other mRNA targets have been previously described for miR-203. For instance, miR-203 reduces p63 in skin differentiation [42], [43], and Akt2, Src, c-jun, survivin and bcl-w in bladder cancer [33], [34], [36], [44]. In addition, in prostate cancer, CKAP2, LASP1, BIRC5, WASF1, ASAP1 and RUNX2 mRNAs were recently identified as new miR-203 targets, suggesting that miR-203 could be a new prognostic marker and a therapeutic target in metastasis of prostate cancer [45].

The antiproliferative function of miRNA-203 was reported a few years ago [46], but the complete set of targets have not been identified, still an obstacle to understand miR-203 function in cellular proliferation is the lack of well established validated targets for specific type of cancer tissues. Our results have identified Hakai as a critical effector of miR-203 actions on cell proliferation (Figure 4 and 5). Supporting this point, the increased cell proliferation seen after lowering miR-203 was strongly dependent on the presence of Hakai, since Hakai silencing abolished the proliferative phenotype (Figure 5C and 5D). miR-203 represses the expression of other proliferative proteins in epithelial tissues, such as survivin or bcl-w [33], [36], so Hakai may function as one of several protein coordinately regulated by miR-203 in order to modulate cell proliferation. Apart from the validated miR-203 targets, there are numerous predicted miR-203 targets waiting for validation; they include mRNAs encoding proteins implicated in the pathways MAPK, Wnt, Notch, or IRS, which may also affect cell proliferation. Therefore, the regulation of Hakai by miR-203, at least, partially contributes to the regulation of cell proliferation.

In this work, we have used epithelial cells that do not express E-cadherin in order to better study the effect of Hakai on cell proliferation. This situation recapitulates the events that occur at early stages of tumour progression. Indeed, Hakai is also detected in tissues that do not express E-cadherin, such in the endoderm epithelia or visceral mesoderm in *Drosophila*, or in human spleen and skeletal muscle [7], [13]. Moreover, Hakai can affect cell proliferation in an E-cadherin-independent manner, suggesting that it may be considered an oncogenic factor [7], [13]–[15]. Furthermore, Hakai was proposed as a correpresor of estrogen receptor alpha (ERα) in breast cancer cells [12], which is according to previously reported ubiquitin-ligases that function as transcriptional regulators [47], [48].

The implication of miR-203 in different malignancies, such as prostate or bladder cancers, was extensively studied in the past [33], [45]. miR-203 was also reported to be epigenetically silenced in hematopoietic malignancies and hepatocellular carcinomas [34], [35]. Here, we investigated its role in colon cancer tissues. Given that Hakai was found up-regulated in human colon adenocarcinomas [6], [14], we extended our study by analyzing miR-203 expression by *in situ* hybridization. We confirmed that Hakai abundance was markedly higher in 17 tumour samples compared with adjacent healthy colon tissues out of 19 pairs analyzed (Figure 6A and Figure S4). By *in situ* hybridization, miR-203 presented lower levels of miR-203 in 3 healthy samples compared to colon cancer from 4 pairs analyzed, supporting the inverse correlation between Hakai expression and miR-203 levels. These findings agree with the hypothesis that miR-203 could help to maintain low levels of Hakai in normal tissues, and that reduced miR-203 levels in colon cancer tissues could contribute to maintain elevated Hakai levels. In light of the influence of miR-203 on Hakai, future studies to test whether miR-203 expression is broadly reduced in cancer are warranted. In light of our results and the tumour suppressive function described for miR-203 in other cancer types [32]–[35], we propose that miR-203 could be a potentially useful prognostic marker and a therapeutic target in colon cancer.

## Supporting Information

Figure S1 **E-cadherin expression in the indicated epithelial cell lines.** Western blot analysis was carried to detect E-cadherin; α-tubulin signals were assessed as loading control.
(EPS)

Figure S2 **Analysis of a microRNA that is not predicted to interact with Hakai mRNA.** A, effect of the indicated transfected pre-miR-21, anti-miR-21 or scrambled Ctrl miRNA on Hakai levels tested in HeLa whole-cell lysates. Analysis was carried out by Western blotting using Hakai antibody and α-tubulin antibody as loading control. The western blotting data are representative of three independent experiments. B, Quantification by densitometry of the Western blotting signals. Values are the means ± SEM from three independent experiments. Student’s T-test analyses indicate no significantly difference relative to scrambled (Ctrl.) miRNA (p>0.25, n = 3).
(EPS)

Figure S3 **Influence of the indicated miRNAs on pEGFP-Hakai constructs.** Forty-eight h after cotransfection of HeLa cells with pEGFP-Hakai construct together with Pre-miR-203, Anti-miR-203 or scrambled Ctrl miRNA, the levels of GFP and α-tubulin (loading control) were measured by Western blot analysis by using anti-GFP and anti-α-tubulin antibodies. Western blotting data are representative of two independent experiments.
(EPS)

Figure S4 **Quantification of Hakai immunohistochemical staining.** Values are the means ± S.E.M of the staining intensity signal scoring per area. Calibration and quantification of the images were performed with AnalySIS^D^ 5.0 software. Mann-Whitney U test analyses show statistical differences in tumour samples respect to paired healthy samples (***p<0.001, n = 19).
(EPS)
